# Is the Grass Always Greener? Comparing the Environmental Impact of Conventional, Natural and Grass-Fed Beef Production Systems

**DOI:** 10.3390/ani2020127

**Published:** 2012-04-10

**Authors:** Judith L. Capper

**Affiliations:** Department of Animal Sciences, Washington State University, Pullman, WA 99164, USA; E-Mail: capper@wsu.edu; Tel.: +1-607-379-9229; Fax: +1-509-335-1082

**Keywords:** beef, carbon footprint, environmental impact, greenhouse gas, productivity, feedlot, corn, grass-fed

## Abstract

**Simple Summary:**

The environmental impact of three beef production systems was assessed using a deterministic model. Conventional beef production (finished in feedlots with growth-enhancing technology) required the fewest animals, and least land, water and fossil fuels to produce a set quantity of beef. The carbon footprint of conventional beef production was lower than that of either natural (feedlot finished with no growth-enhancing technology) or grass-fed (forage-fed, no growth-enhancing technology) systems. All beef production systems are potentially sustainable; yet the environmental impacts of differing systems should be communicated to consumers to allow a scientific basis for dietary choices.

**Abstract:**

This study compared the environmental impact of conventional, natural and grass-fed beef production systems. A deterministic model based on the metabolism and nutrient requirements of the beef population was used to quantify resource inputs and waste outputs per 1.0 × 10^9^ kg of hot carcass weight beef in conventional (CON), natural (NAT) and grass-fed (GFD) production systems. Production systems were modeled using characteristic management practices, population dynamics and production data from U.S. beef production systems. Increased productivity (slaughter weight and growth rate) in the CON system reduced the cattle population size required to produce 1.0 × 10^9^ kg of beef compared to the NAT or GFD system. The CON system required 56.3% of the animals, 24.8% of the water, 55.3% of the land and 71.4% of the fossil fuel energy required to produce 1.0 × 10^9^ kg of beef compared to the GFD system. The carbon footprint per 1.0 × 10^9^ kg of beef was lowest in the CON system (15,989 × 10^3^ t), intermediate in the NAT system (18,772 × 10^3^ t) and highest in the GFD system (26,785 × 10^3^ t). The challenge to the U.S beef industry is to communicate differences in system environmental impacts to facilitate informed dietary choice.

## 1. Introduction

Sustainability is often defined as “*meeting society’s present needs without compromising the ability of future generations to meet their own needs*” and comprises three interlinked facets: environmental responsibility, economic viability and social acceptability [1]. In this context, the sustainability of beef production comes under considerable scrutiny. Global food security and environmental issues are significant considerations for governments and policy-makers who are conscious not only of the proportion of their national population that is currently food-insecure, but also of the prediction that the global population will increase to over 9.5 billion people by the year 2050 [2]. The greatest population increases are predicted to occur in developing regions such as Africa, China and India, and, by 2050, these nations are predicted to enjoy a per capita income similar to that currently seen within Europe and North America [3]. As incomes increase, so does the demand for high-quality animal proteins such as meat, milk and eggs, thus the Food and Agriculture Organization of the United Nations (FAO) suggests that food requirements will increase by 70% by 2050 [2]. In the event of considerable population growth, future competition for water, land and energy between livestock production and human activities will increase. The global beef industry will therefore face a significant challenge in fulfilling consumer demand for meat products, using a finite resource base. This issue is not confined to a future scenario—current concern over dwindling natural resources, climate change and the social acceptability of beef production practices leads to debate as to whether the U.S. beef industry should continue to intensify and improve productivity to feed the increasing population, or adopt extensive production systems often perceived by consumers to have a lower environmental impact [4]. 

Advances in nutrition, genetics and management have conferred considerable advances in reducing the environmental impact of beef production over time: Capper [5] demonstrated that compared to beef production systems characteristic of 1977, modern beef production in 2007 used 19% less feed, 12% less water, 33% less land and exhibited a 16% decrease in the carbon footprint per unit of beef. The improvements in efficiency conferred by modern management practices and technology use facilitate the production of economically-affordable beef [6,7]. Nonetheless, the social acceptability of specific beef production practices, specifically finishing within feedlots and the use of technology to improve growth rate, may be perceived as undesirable by the consumer due to concerns relating to animal welfare [8,9], human health [10] or environmental sustainability [11]. Beef produced without the use of growth-enhancing technology (GET; “natural” beef), or finished on a forage-based diet (“grass-fed”) may therefore gain market share [12]. The aim of this study was to evaluate the comparative environmental impacts (defined as resource use and greenhouse gas (GHG) emissions) of conventional, natural and grass-fed beef production using a deterministic whole system model based on ruminant nutrition and metabolism. 

## 2. Experimental Section

This study utilized data from existing reports and databases and required no Animal Care and Use Committee approval. A deterministic environmental impact model (EIM) based on the nutrient requirements and metabolism of animals within all sectors of the beef production system was used to quantify the environmental impact of three U.S. beef production systems: “Conventional”, (CON) “Natural” (NAT) and “Grass-fed” (GFD). The CON system represented the beef production system characteristic of the majority of beef operations. A myriad of definitions exist for “natural” beef, thus in this study management practices in the CON and NAT system were identical, save for the use of GET in the CON system (where approved by the FDA) at 100% adoption rate, compared to zero adoption in the NAT system. The GFD system was defined by the USDA-AMS standard for grass-fed beef [13], which prescribes a forage-based diet from birth to slaughter without grain or other non-forage supplementation. No prohibition of GET exists in the USDA-AMS standards for grass-fed beef production, but such technologies are seldom compatible with marketing claims for grass-fed beef, therefore they were not employed in the current comparison. Environmental impact was calculated by comparing annual resource inputs and waste output of each beef production system, expressed per 1.0 × 10^9^ kg of beef (hot carcass weight) produced in 365 d.

### 2.1. The Beef Production System Environmental Model

**Figure 1:**
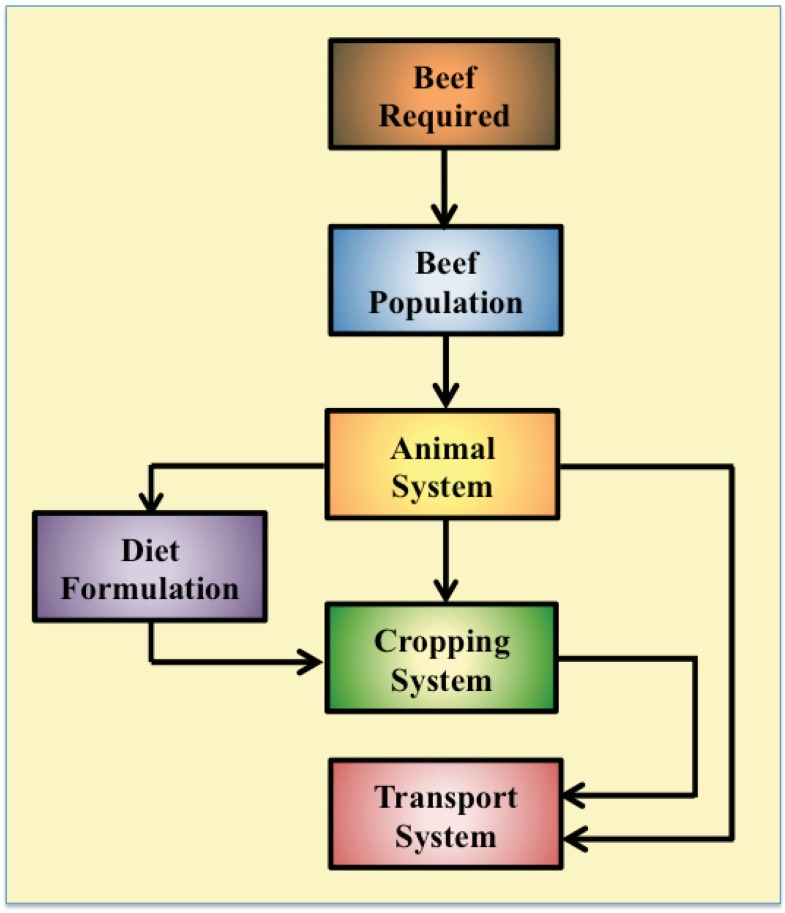
Simplified schematic representation of the sub-systems within the environmental impact model.

A deterministic EIM of beef production was created within Microsoft Excel. The EIM contained four different sub-models: the beef population, the animal system, the cropping system and the transportation system (Figure 1). 

#### 2.1.1. The Beef Population Sub-Model

**Figure 2:**
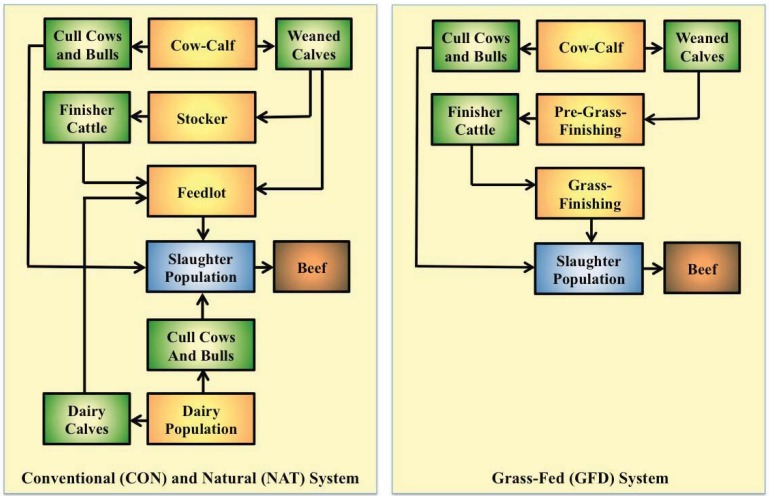
Schematic representation of the animal systems modeled within the study.

The model worked step-wise backwards through the production chain. The functional unit (1.0 × 10^9^ kg of hot carcass weight beef) and the slaughter characteristics of the various beef populations determined the number of slaughter animals required and thus the total beef population size. The numbers of animals within each of the six sub-systems (cow-calf unit, stocker operation, pre-grass-finishing system, feedlot, grass-finishing system and dairy population) contained within the animal system sub-model (ASSM; Figure 2) were derived from the total slaughter population size according to sub-system-specific productivity metrics (mortality, growth rate) as detailed in Section 2.2 and Section 2.3, and pro-rated on an annual basis according to the number of days spent within each system.

#### 2.1.2. The Animal System Sub-Model

The ASSM contained six sub-systems. The cow-calf unit contained beef breed animals (Angus cows, Hereford bulls and Angus × Hereford offspring) that served to support population dynamics (lactating and dry cows, pre-weaned calves, replacement heifers, adolescent bulls, yearling bulls and mature bulls). The stocker operation and pre-grass-finishing system contained weaned beef breed steers and heifers fed until they reached sufficient weight to be placed into the feedlot or the grass-finishing system. The feedlot contained calf-fed beef and dairy (Holstein) animals that enter the feedlot at weaning (beef calves) or four months of age (dairy calves); and yearling-fed beef breed animals that enter the feedlot after the stocker stage. Within the feedlot, cattle were fed until the desired weight and condition were achieved. A dairy system was also contained within the CON and NAT systems for the purposes of supplying dairy-bred calves and cull dairy cows and for consequent allocation of resources and emissions between the beef and dairy system. The grass-finishing system contained beef breed steers and heifers fed until the desired weight and condition was achieved.

Nutrient requirements and feed intakes for each class of animals were calculated using AMTS Cattle Pro [14], a commercial cattle diet formulation software package based on the Cornell Net Carbohydrate and Protein System. Diets were formulated to fulfill the requirements of animals within each class and sub-system according to age, sex, breed, liveweight, average daily gain, production system characteristics and GET use (where appropriate) to within 1% of predicted metabolizable energy and protein requirements. Outputs from AMTS Cattle Pro (growth rate, DMI, dietary composition, dietary fiber characteristics, manure output, N and P excretion) were then inputted into the ASSM. Voluntary water intake for mature cows was modeled according to Beckett and Oltjen [15], with water intakes for all other classes of animal calculated from the equation derived by Meyer *et al.* [16]. Total manure output, N and P excretion were calculated as the sum totals from each animal class within the ASSM expressed per function unit of output (1.0 × 10^9^ kg beef). Total carbon emissions from the ASSM comprised CH_4_ and N_2_O from both enteric fermentation and manure. Dietary soluble residue, hemicellulose and cellulose intakes were used to calculate enteric CH_4_ production from all animals within each sub-system, including pre-weaned calves [17]. The fraction of nitrogen emitted as enteric N_2_O was modeled using data reported by Kaspar and Tiedje [18] and Kirchgessner *et al.* [19]. Emissions of CH_4_ from manure were estimated using methodology prescribed by the U.S. Environmental Protection Agency [20] based on the quantity of volatile solids excreted, maximum CH_4_-producing potential (0.24 cubic meters per kg of volatile solids), and a conversion factor specific to either pasture or feedlot systems. Intergovernmental Panel on Climate Change [21] emission factors were used to calculate N_2_O emissions from manure. 

#### 2.1.3. The Cropping System Sub-Model

Quantities of feedstuffs required to support beef production were derived from the ASSM according to dietary formulation, daily DMI for each animal class and animal numbers. Land use was calculated according to feedstuff requirements and cropping yields. Crop yields and inputs (fertilizers, herbicides, insecticides, fuel use) were derived as detailed by Capper [5]. Irrigation water use for crop production was calculated from application rates and proportions of crops irrigated according to the Census of Agriculture Ranch and Irrigation Survey [22]. Carbon emissions from feedstuff production comprised N_2_O and CO_2_ from crop production expressed as CO_2_-equivalents. Emissions of N_2_O from fertilizer application, manure application to crops, and manure applied while grazing were estimated from the factors published by the IPCC [21]. Emissions of CO_2_ from fertilizer and pesticide manufacture were derived from West and Marland [23], and similar emissions from fossil fuel combustion for crop production were calculated from US EPA [24]. Biogenic carbon, which rotates continuously through a cycle comprising uptake of atmospheric carbon by crops followed by a return to the atmosphere through animal respiration, was considered to be neutral with respect to GHG emissions. Carbon sequestration into soil and CO_2_ produced through animal respiration were considered to be equivalent and were therefore not specifically accounted for.

#### 2.1.4. The Transportation Sub-Model

The assumptions underlying the transportation sub-model are described within Capper [5]. Carbon emissions from animal transport were derived from animal numbers, the carrying capacity of haulage vehicles dependent upon animal liveweight and vehicle size, distances between animal sub-systems and fuel efficiency. Within the CON and NAT systems, animals were transported an average of 483 km between sub-systems and 161 km to the slaughterhouse. Animal transport within the GFD system was confined to the transport of animals from the grass-finishing system to the slaughterhouse (161 km) as animals were assumed to stay within the same farm premises from birth to slaughter. Within the CON and NAT systems, feed (corn and soy) was transported 558 km to the feedlot (underlying assumptions described by Capper [5]), with carbon emissions dependent upon feedstuff requirements, vehicle carrying capacity and fuel efficiency. All forages within the GFD system were assumed to be home-grown, thus no feed transport costs were assigned to this system.

### 2.2. Conventional and Natural Beef Production System Characteristics

The CON and NAT beef production systems comprised cow-calf, stocker and feedlot operations modeled according to characteristic U.S. production practices [25,26,27,28] with population characteristics unaffected by GET use as detailed in Capper [5]. Briefly, these included a 365 d calving interval, a 207 d lactation, and a calving rate of 91.5% with 96.5% of cows producing a live calf. Replacement heifers were included in the population at a rate of 0.27 heifers per cow with an annual replacement rate of 12.9% and a 24-month age at first calving. Bulls were included in the population at a ratio of one bull per 25 cows.

Diets for animals in the CON and NAT supporting populations (lactating and dry cows, replacement heifers, mature and adolescent bulls) were formulated based on pasture, grass hay and straw, adjusted for a predominantly pasture-based diet during spring and summer, with conserved forage supplementation during fall and winter. Prior to weaning at 207 d [26], calves suckled from the dam and consumed pasture and starter feed (flaked corn and soybean meal). Post-weaning, 83.5% of calves [5] entered the stocker sub-system where they were fed primarily pasture-based diets with supplemental grass hay, corn silage, flaked corn and soybean meal according to seasonal pasture availability. At 386 kg bodyweight (BW; steers) or 340 kg BW (heifers), stocker cattle entered the feedlot as yearling-fed finishing animals. 

Diets for yearling-fed feedlot steers and heifers were balanced for predicted dry matter intake (DMI) and growth rates (Table 1) and comprised corn grain, soybean meal, alfalfa hay and vitamin/mineral supplements. A total of 16.5% [5] of weaned beef calves entered the feedlot directly as calf-fed finishing animals and were fed a diet containing the same base ingredients as the yearling-fed animals, formulated for predicted DMI and average growth rates as documented in Table 1. 

The CON and NAT beef systems included animal inputs from the U.S. dairy industry in terms of cull cows, plus male and dairy female calves at 3 d of age. The question of resource and waste allocation between interlocking systems (in this case the dairy and beef populations) has been extensively debated [29]. Resource inputs and waste output between the dairy and beef systems were calculated based upon a biological allocation method. A deterministic model of resource use and environmental impact within dairy production was previously developed by Capper *et al.* [30], based upon the same nutrition and metabolism principles as the current beef model. Employing the model described by Capper *et al.* [30] to assess the environmental impact of dairy inputs to the beef industry within the current study ensured that resource input data for both models were sourced from similar data, thus minimizing methodological conflict between the models. The dairy model was used to determine the proportion of total resource inputs and waste output attributable to growth in Holstein heifers from birth up to 544 kg (the weight at which they would be sold as beef animals if they did not enter the dairy herd). These totals represented the environmental cost attributed to dairy cull cows entering the beef market and were added to the appropriate beef production system according to the number of cull cows within said system. The impact of producing male and female dairy calves for calf-fed rearing was calculated by partitioning out the proportion of total resource inputs and waste output attributable to pregnancy in lactating and dry dairy cows. This cost was adjusted for the number of dairy calves in the beef system and thus the number of cows required, before application to the beef production system. Use of this allocation method ensured that the dairy industry was credited for by-product animals that were ultimately destined to produce meat within the beef production system.

**Table 1:** Mean production data for sub-classes of growing and finishing animals within three beef production systems: conventional (CON), natural (NAT) or grass-fed (GFD) ^a^.

	System	Time in sub-system (d)	Growth rate (kg/d)	Weight change (kg)	End weight (kg)	Slaughter data	
						Age (d)	Weight (kg)
Pre-weaned beef calf	CON	207	0.98	203	245	N/A	N/A
	NAT	207	0.98	203	245	N/A	N/A
	GFD	207	0.88	183	226	N/A	N/A
Pre-weaned dairy calf ^b^	CON	56	0.92	51	92	N/A	N/A
	NAT	56	0.92	51	92	N/A	N/A
Stocker	CON	123	0.99	122	367	N/A	N/A
	NAT	159	0.77	122	367	N/A	N/A
Pre-grass finishing	GFD	159	0.42	67	293	N/A	N/A
Calf-fed beef in feedlot	CON	203	1.61	326	571	410	571
	NAT	203	1.20	244	489	435	489
Calf-fed dairy in feedlot	CON	259	1.74	449	541	315	541
	NAT	259	1.48	383	476	315	476
Yearling-fed beef in feedlot	CON	110	1.86	204	571	440	571
	NAT	110	1.48	163	530	440	530
Grass-finished	GFD	313	0.61	192	486	679	486

^a^ Further details of system characteristics are given in Section 2. ^b^ Although calves were weaned at 56 days, they remained on the calf ranch until 120 days of age. The 64-day post-weaning period has been incorporated into the calf-fed dairy phase for ease of understanding.

A total of 12.9% of animals within the CON and NAT feedlot finishing systems originated from dairy production, comprising 11.5% dairy steers and 1.4% dairy heifers [5,15]. Within the model, dairy calves were fed milk replacer and a calf starter ration (flaked corn and soybean meal) until weaning at 56 d. Dairy calves entered the feedlot on a calf-fed basis and were finished on a standard feedlot diet similar to that fed to the calf-fed beef animals, balanced for predicted DMI and growth rate (Table 1). 

The CON system included the use of GET in terms of steroid implants, in-feed ionophores (monensin sodium, lasalocid sodium) in-feed hormones (melengestrol acetate, MGA) and beta-adrenergic agonists (ractopamine hydrochloride, zilpaterol hydrochloride, βAA). Ionophore use in lactating and dry beef cows was modeled according to Sprott *et al.* [31] with a 10.2% reduction in feed intake while maintaining performance. AMTS Cattle Pro [14] has a built-in module within the software that corrects feed intake, efficiency and growth rate for the use of steroid implants and ionophores in growing cattle, therefore this was employed when formulating diets for stocker and feedlot animals. Due to a lack of data for the effects of implant use in pre-weaned calves and the characteristically low adoption rate of this technology within this animal class [25], this technology was not included in the pre-weaned calf groups. The effects of MGA use in heifers were modeled according to data from Perrett *et al.* [32] and Sides *et al.* [33,34,35,36,37] that showed a central tendency towards a 3.5% increase in feed intake compared to non-supplemented animals. Research relating to the productivity effects of βAA demonstrated a central tendency to increase growth rate by 18.4% during the supplementation period (28 d for ractopamine hydrochloride, 20 d for zilpaterol hydrochloride) across all classes of supplemented animal [34,35,36,37,38,39,40,41,42,43,44]. The dressing percentage for animals supplemented with βAA (CON) averaged 63.8% compared to 63.3% for non-supplemented animals (NAT) [34,35,36,37,38,39,42,43,44,45].

Slaughter populations for the CON and NAT systems comprised calf-fed and yearling-fed beef-breed animals; calf-fed dairy animals and cull animals from the beef and dairy sectors. Sub-classes of feedlot-finished animals were taken to the same number of days on feed within both models, for example, 110 days on feed for yearling-fed beef steers in both the CON and the NAT systems, as shown in Table 1. The average slaughter weight across all animal categories was 569 kg in the CON system and 519 kg in the NAT system, at average ages of 444 d (CON) and 464 d (NAT). 

### 2.3. Grass-Fed Beef Production System Characteristics

The GFD production system included a cow-calf operation, a pre-grass-finishing operation and a grass-finishing operation. Supporting population characteristics unaffected by technology use (calving interval, age at first calving, calving rate, lactation length, replacement heifer:cow ratio and bull:cow ratio) were as detailed in Capper [5] and briefly described in Section 2.2. 

All animals in the GFD system were supplied with a forage-based diet formulated based on pasture, alfalfa hay, grass hay and wheat straw, adjusted for a pasture-based diet during spring and summer, with conserved forage supplementation during fall and winter. All diets were formulated according to AMTS Cattle Pro [14] based on predicted DMI and growth rate. Prior to weaning at 207 d, calves suckled from the dam and consumed pasture. Post-weaning, all weaned calves entered the pre-grass-finishing sub-system where they were fed pasture, alfalfa hay and grass hay diets according to seasonal pasture availability. Cattle entered the grass-finishing system at 12 mo of age to coincide with spring grass availability. 

As dairy calves entering the beef system are characteristically finished within feedlots and cull dairy cows would not be eligible to be sold as grass-fed beef, the GFD system did not include any animals from the dairy industry. Slaughter populations for the GFD system therefore comprised grass-finished steers and heifers, plus cull beef breed cows and bulls. The average slaughter weight across all animal categories was 486 kg at 679 d of age, with a 57.5% dressing percentage.

## 3. Results and Discussion

Productivity is a major driver of environmental impact via the “dilution of maintenance” effect [5]. This concept is demonstrated by the results of the current study. Animals within the CON system had an average slaughter weight of 569 kg and took a total of 444 d to raise from birth to slaughter; compared to 519 kg slaughter weight per animal after a similar time period (464 d) in the NAT system; and 486 kg after 679 d in the GFD system. As slaughter weight increases, concurrent decreases are exhibited in the number of finished beef animals required to produce a set quantity of beef, and the number of non-productive animals required to maintain the supporting beef population. Thus, the CON system required 7,046 × 10^3^ animals in the population to produce 1.0 × 10^9^ kg of beef compared to 8,257 × 10^3^ animals (a 17.1% increase) and 12,510 × 10^3^ animals (a 77.5% increase) in the NAT and GFD systems respectively (Table 2). 

Improvements in growth rate do not necessarily affect the size of the supporting beef population; however, the time elapsing from birth to slaughter has a notable effect upon the total population maintenance nutrient requirement. It is important to note that the growth rates within this study are those predicted by the AMTS Cattle Pro [14] ration formulation software based on animal characteristics and dietary nutrient supply, and are not representative of any specific farm. Animal productivity varies considerably between and within individual systems, and it could be argued comparisons between individual farms might show differing results than those exhibited in the current study. The average time from birth to slaughter in the GFD system (679 d) is considered to be a conservative estimate as it is at the lower end of the range of finishing ages (671–915 d) quoted during personal communication with a grass-fed beef producer, Joel Salatin, Polyface Farm, Swoope, VA, USA, who is noted for a highly-successful forage-based system. 

As shown in Table 2, reducing slaughter weight and growth rate increases the population nutrient requirement of the CON system (228,651 × 10^6^ MJ ME) by 11.5% in the NAT system (254,841 × 10^6^ MJ ME) or 54.6% in the GFD system (353,484 × 10^6^ MJ ME). The population maintenance nutrient requirement can be considered a proxy for both resource use and GHG emissions [5], thus, as shown in Table 2, environmental impact measured as a function of any measured parameter was reduced in the CON system compared to the NAT or GFD system. These results concur with those of a previous study evaluating the ecological impact of beef technology use and production system [46], which demonstrated considerable decreases in land use and methane emissions, and increased habitat conservation in an intensive system compared to a grass-fed system. Moreover, Pelletier [47] compared of various beef finishing systems using partial life cycle assessment, concluding that the greatest environmental impact was conferred by extensive grass-finishing systems compared to intensive feedlot-finishing systems; with the lowest impact bestowed by systems with the shortest time interval from birth to slaughter (calf-finished beef production).

**Table 2:** Resource inputs, waste output and environmental impact associated with producing 1.0 × 10^9^ kg of beef from a conventional (CON), natural (NAT) or grass-fed (GFD) system ^a^.

System	CON	NAT	GFD
Animals			
Supporting population ^b^ (×10^3^)	5,539	6,265	8,482
Stockers/Pre-finishing (×10^3^)	628	920	1,378
Finishing animals (×10^3^)	2,334	2,640	3,045
Total animals slaughtered ^c^ (×10^3^)	2,756	3,117	3,580
Total population ^d^ (×10^3^)	7,046	8,257	12,510
Nutrition resources			
Population energy requirement ^e^ (MJ × 10^6^)	228,651	254,841	353,484
Feedstuffs (t × 10^3^)	54,476	67,263	106,166
Land (ha × 10^3^)	5,457	6,678	9,868
Water (liters × 10^6^)	485,698	572,477	1,957,224
Fossil fuel energy (MJ × 10^6^)	8,773	10,304	12,290
Waste output			
Manure (t × 10^3^)	36,976	45,431	74,392
Nitrogen excretion (t)	399,789	486,683	807,759
Phosphorus excretion (t)	37,190	46,897	76,567
Greenhouse gas emissions			
Methane ^f^ (t)	501,593	586,729	854,561
Nitrous oxide ^g^ (t)	7,532	9,078	13,833
Carbon footprint ^h^ (t CO_2_-eq × 10^3^)	15,989	18,772	26,785

^a^ Further details of system characteristics are given in Section 2. ^b^ Includes cows (lactating and dry), pre-weaning calves, heifers (<12 mo and >12 mo of age) and bulls (adolescent, yearling and mature). ^c^ Includes cull cows and bulls. ^d^ Total is not equivalent to the sum of the previous rows due to differences in mortality between sub-systems. ^e^ Includes energy requirements for maintenance (all animals), and growth, pregnancy and lactation (where applicable) ^f^ Includes CH_4_ emissions from enteric fermentation and manure. ^g^ Includes N_2_O emissions from manure and inorganic fertilizer application. ^h^ Includes CO_2_ emissions from manufacture of cropping inputs, crop production and harvest, fuel combustion, electricity generation, and CO_2_ equivalents from CH_4_ and N_2_O.

Following established historical trends, the quantity of arable land available per capita is predicated to decrease in accordance with the global population size, reaching a nadir at 0.15 ha/person in 2050 [48]. This is a consequence of increased demand for land used for non-agricultural purposes (e.g., industry, recreation, urban sprawl) and degradation of existing agriculture land [49]. Efficient land use is crucial for agricultural sustainability, thus the CON system, which required 5,457 × 10^3^ ha of land per 1.0 × 10^9^ kg beef, appears to be more sustainable than either the NAT system which required 22.4% more land (6,678 × 10^3^ ha of land per 1.0 × 10^9^ kg beef) or the GFD system at 80.8% more land to produce the same quantity of beef (9,868 × 10^3^ ha of land per 1.0 × 10^9^ kg beef; Table 2). Existing debate as to the validity of using grains or legumes for animal feed that could be otherwise be used for human food [50,51] is likely to intensify as the population increases. For example, despite its biological implausibility, a feed efficiency of 30 kg feed to one kg gain has recently been quoted as evidence of the unsustainability of beef production [52]. Monogastric animals have an improved efficiency of feed conversion into gain compared to ruminants. However, beef production systems that utilize range and pastureland (which is generally unsuitable for human food crop production [5]) gain a sustainability advantage over monogastric production systems that rely upon human-edible grains and legumes. This is discussed at length by Wilkinson [53], who redefined the conventional measures of feed efficiency (7.8 kg feed per kg of gain for feedlot-finished beef) to account for the human-edible energy or protein feed inputs compared to the human-edible energy or protein output from the animal production system. Under these constraints, grass-finished beef (termed suckler beef in European systems) had a favorable human edible feed efficiency ratio whether expressed in terms of energy (1.9 MJ/MJ edible energy in animal product) or protein (0.92 kg/kg edible protein in animal product). Wilkinson’s [53] results appear to imply that grass-fed beef would be environmentally advantageous if competition for feed/food crops is a defining criteria, however, the quantity of land required for differing production systems must be taken into consideration. If the total U.S. beef produced in 2010 (11.8 × 10^9^ kg) was produced by a grass-fed system, the increase in land required compared to conventional production would be 52.2 × 10^6^ hectares, equivalent to 75% the land area of Texas. 

Water use for agriculture is an area of growing concern within many regions and is predicted to be the primary limiting factor affecting agricultural productivity in future [54] as agricultural requirements conflict with industrial and urban use, and the rate of withdrawal from aquifers exceeds replenishment. Within beef production, water is used within two major sub-systems: the animal sub-system in terms of voluntary water intake, and the cropping sub-system, in which water is used for crop and pastureland irrigation. As with other environmental measures, animal productivity has a considerable effect on water consumption as a maintenance requirement for water may be partitioned out for each individual animal. Thus increased growth rate and slaughter weight in the CON system reduces water consumption to 485,689 × 10^6^ liters (CON) compared to a 17.9% increase in the NAT system (572,477 × 10^6^ liters per 1.0 × 10^9^ kg beef) or a 302% increase in the GFD system (1,957,224 × 10^6^ liters per 1.0 × 10^9^ kg beef; Table 2). However, irrigation water is the major contributor to total water consumption, thus the magnitude of the difference in water use between the CON and GFD systems (compared to the proportional differences in other environmental measures) is due to the assumption within the model that 50% of grassland used to finish cattle in the GFD system is irrigated. This is an area of uncertainty compared to the irrigation data for the feed crop (corn, soy, alfalfa) components of the model. USDA irrigation surveys [22] provide data upon average water use per pastureland unit area and the percentage of pastureland irrigated on a national basis, yet there is no data available as to how much irrigated pastureland is allocated to beef. If we change the original assumption (50% of pastureland used to finish cattle is irrigated) and run the model with 25%, 15% or 5% of land being irrigated, the total quantity of water used by the GFD system declines from 1,957,224 × 10^6^ liters to 1,044,070 × 10^6^ liters (25%), 678,808 × 10^6^ liters (15%) or 313,547 × 10^6^ liters (5%). Thus, the model is sensitive to irrigation water use to the extent that if greater than 9.7% of land used to finish beef is irrigated (while holding irrigation water use within the CON system constant), the GFD system is less environmentally-desirable than the CON system.

Nutrient (N and P) excretion was primarily affected by animal productivity (Table 2), with minor effects of nutrient supply *vs*. requirements. The quantities of N and P excreted from the population per 1.0 × 10^9^ kg kg beef were reduced in the CON system (399,789 t N/kg beef and 37.190 t P/kg beef) compared to the NAT system (486,683 t N/kg beef and 46,897 t P/kg beef) or GFD system (807,759 t N/kg beef and 76,567 t P/kg beef). Nutrient run-off into waster courses is a primary concern relating to P excretion, and N excretion is also associated with ammonia emissions to the atmosphere, particularly in confined animal systems. Variation in manure application rate, storage characteristics, climatic conditions and pasture-based/housed animal management will have a considerable effect upon both nutrient run-off [55] and ammonia emissions [56]. It should therefore be noted that neither P nor N excretion provides a direct measure of nutrient run-off or ammonia emissions, but simply act as a comparative measure for the potential for run-off or gaseous emissions to occur.

The carbon footprint (expressed as total GHG emissions in CO_2_-equivalents per unit of beef) of livestock production systems is one of the most debated issues relating to environmental impact. Previous research has demonstrated that improving productivity demonstrably reduces the carbon footprint of beef production [5,47,57,58,59,60], which concurs with the results revealed by the 17.4% increase in NAT system carbon emissions (18,772 t CO_2_-eq per 1.0 × 10^9^ kg beef) compared to the CON system (15,989 t × 10^3^ CO_2_-eq per 1.0 × 10^9^ kg beef; Table 2) within the current study. Nonetheless, the perception remains that extensive, grass-based systems have a lower carbon footprint than intensive, confined systems. This is exemplified by a report from the Environmental Working Group [61] that states “Meat, eggs and dairy products that are certified organic, humane and/or grass-fed are generally the least environmentally damaging.” Within the current study, the GFD system had a carbon footprint of 26,785 t CO_2_-eq per 1.0 × 10^9^ kg beef, which, is an increase of 67.5% compared to the CON system and would be equivalent to adding 25.2 × 10^6^ US cars to the road on an annual basis based on average mileages and carbon emissions per mid-sized automobile from US EPA [24]. The increase in carbon emissions was primarily affected by the increase in population size and time elapsed from birth to slaughter in the GFD population, however, provision of a forage-based diet also increased daily methane emissions per animal as noted by Johnson and Johnson [62] and Pinares-Patiño *et al.* [63]. 

The potential for carbon sequestration by well-managed pastureland may be a mitigating factor for carbon emissions within the GFD system, yet it was not accounted for throughout the current study due to a lack of sustentative data. Although the GFD system is forage-based throughout, the cow-calf and stocker sub-systems within the CON and NAT production systems were also forage-based. In the absence of significant differences in land conversion or management in these sub-systems, potential for carbon sequestration could therefore only be considered to be a mitigating factor within the grass-finishing system compared to the feedlot-finishing sub-system. Partitioning out the carbon emissions from sub-systems reveals that the grass-finishing sub-system accounted for 6,868 t × 10^3^ CO_2_-eq per 1.0 × 10^9^ kg beef. With a total land use of 1,392 × 10^3^ ha in the grass-finishing sub-system and assuming carbon equilibrium for land used by the feedlot-finishing system, the pastureland used to finish cattle in the GFD system would need to sequester 4.93 t CO_2_ per ha/yr, equivalent to 1.35 t C per ha/yr, in order to produce a finishing sub-system with a similar carbon footprint to that of the CON system. This appears to be a lofty target, given that Bruce *et al.* [64] suggest that the potential for carbon sequestration in well-managed pastureland is 200 kg/ha, whereas Conant *et al.* [65] report 540 kg/ha. Moreover, this does not take into consideration the increased land use and carbon emissions from cow-calf and stocker populations in the GFD compared to the CON system. As cow-calf and stocker operations tend to be located on unimproved rangeland or forage crops that do not achieve significant carbon sequestration [64], the estimate of the amount of carbon needed for the GFD system to reach equal carbon emissions per unit of beef should be regarded as a considerable underestimate. Well-managed rotational grazing systems within the cow-calf operation would lessen the impact of the cow-calf sub-system on total carbon emissions per unit of beef, however, this mitigation is not confined to GFD systems and could equally be practiced within the CON or NAT systems.

Feed and animal transportation are often considered to be a major factor affecting fossil fuel use in CON or NAT beef production systems, yet within the current study transport accounted for 0.87% of the carbon footprint from the CON system, 0.83% of the NAT system’s carbon emissions and 0.24% of total carbon emitted from the GFD system, a result which is in agreement with the results published by Capper [5]. The increased contribution of transportation to the CON and NAT systems’ carbon footprints resulted from the greater reliance upon feeds imported into the feedlot system, compared to increased proportional contributions of CH_4_ emissions in the GFD system. Fossil fuel use within the three systems followed a similar pattern to the previously discussed resources, with CON system using less fossil fuel energy per 1.0 × 10^9^ kg beef (8,773 × 10^6^ MJ) compared to the NAT (10,304 × 10^6^ MJ, an increase of 17.5%) or GFD (12,290 × 10^6^ MJ, an increase of 40%) systems. This is contrary to the popular belief that lesser fossil fuel use is a major environmental advantage of extensive beef production systems. Within the current study, cropping and harvesting practices are the major contributors to fossil fuel use: decreases in total feed use and therefore cropping inputs and feed transportation resulting from improved animal productivity are demonstrated by the difference in fossil fuel energy between the CON and NAT systems. The greater use of fossil fuel energy in the GFD system results from cropping and harvesting practices for conserved forages to support animals during winter months.

## 4. Conclusions

The US beef industry faces a clear challenge in supplying the needs of the increasing population, while reducing environmental impact. Use of technologies that improve animal productivity in combination with intensive feedlot finishing systems demonstrably reduce both resource use and GHG emissions per unit of beef. The beef industry is thus well placed to continue its tradition of environmental stewardship, yet it faces considerable opposition in terms of consumer perceptions of intensive production systems that may have a negative impact upon social sustainability. Demonization of specific sectors in favor of niche markets that intuitively appear to have a smaller carbon footprint further propagate the idea that large-scale production systems are undesirable, yet all systems that fulfill the three facets of sustainability have a place within the industry. It is important to communicate the relative environmental impacts of differing beef production systems to producers, processors and retailers in order to maintain a variety of beef products within the marketplace and to provide consumers with a scientific basis for dietary choices.
